# Subthalamic nucleus detects unnatural android movement

**DOI:** 10.1038/s41598-017-17849-2

**Published:** 2017-12-19

**Authors:** Takashi Ikeda, Masayuki Hirata, Masashi Kasaki, Maryam Alimardani, Kojiro Matsushita, Tomoyuki Yamamoto, Shuichi Nishio, Hiroshi Ishiguro

**Affiliations:** 1 Research Center for Child Mental Development, Kanazawa University, Kanazawa, Japan; 2 Endowed Research Department of Clinical Neuroengineering, Global Center for Medical Engineering and Informatics, Osaka University, Suita, Japan; 3 Department of Neurosurgery, Osaka University Medical School, Suita, Japan; 4 Center for Information and Neural Networks (CiNet), National Institute of Information and Communications Technology, and Osaka University, Suita, Japan; 5 Graduate School of Engineering Science, Osaka University, Toyonaka, Japan; 6 Institute of Liberal Arts and Sciences, Nagoya University, Nagoya, Japan; 7 Graduate School of Arts and Sciences, The University of Tokyo, Tokyo, Japan; 8 Graduate School of Engineering, Gifu University, Gifu, Japan; 9 Advanced Telecommunications Research Institute International, Kyoto, Japan

## Abstract

An android, i.e., a realistic humanoid robot with human-like capabilities, may induce an uncanny feeling in human observers. The uncanny feeling about an android has two main causes: its appearance and movement. The uncanny feeling about an android increases when its appearance is almost human-like but its movement is not fully natural or comparable to human movement. Even if an android has human-like flexible joints, its slightly jerky movements cause a human observer to detect subtle unnaturalness in them. However, the neural mechanism underlying the detection of unnatural movements remains unclear. We conducted an fMRI experiment to compare the observation of an android and the observation of a human on which the android is modelled, and we found differences in the activation pattern of the brain regions that are responsible for the production of smooth and natural movement. More specifically, we found that the visual observation of the android, compared with that of the human model, caused greater activation in the subthalamic nucleus (STN). When the android’s slightly jerky movements are visually observed, the STN detects their subtle unnaturalness. This finding suggests that the detection of unnatural movements is attributed to an error signal resulting from a mismatch between a visual input and an internal model for smooth movement.

## Introduction

Humanoid technology has made significant advancements towards the production of humanoid robots with human-like capabilities. Although humanoid robots have human-like capabilities, when their human-likeness exceeds a certain threshold, the human’s affinity for them decreases and an uncanny feeling about them increases. This phenomenon is called the ‘uncanny valley.’ The uncanny feeling towards almost human but not fully human-like robots may be produced by different factors^1^. The uncanny valley was originally hypothesized in the 1970s, and it has since been expected that both the appearance and the movement of human-like robots can modulate the observer’s uncanny feeling about them^2^.

The effect of appearance on an uncanny feeling has been a focus of study for many years. The effect can be explained by the categorical boundary of human and non-human agents^3^. An fMRI study has shown that a computer-generated non-human avatar face that was primed for human-likeness activated the putamen, thalamus, caudate nucleus, and red nucleus^4^. This finding implies that priming for human likeness with regard to appearance induces the activation of subcortical nuclei.

The effect of movement on an uncanny feeling was relatively unexplored, but at least two types of explanations for it have been proposed in the recent literature. The first type of explanation is that an uncanny feeling arises from a disconnection between appearance and movement. A robot with a human-like appearance is expected to move in a human-like manner, but it is only able to move in a mechanical manner. An fMRI study found that an uncanny feeling derives from a mismatch between expected and actual movement, and this finding was used to suggest a predictive coding model of action perception^5^. The second type of explanation is that the lack of motion naturalness is important for uncanny feelings. Past studies have shown that an uncanny feeling systematically decreased when an agent acted naturally and that the decrease of an uncanny feeling was independent of the appearance of the agent^6,7^. Accordingly, an uncanny feeling may arise from the lack of human-like motion quality or motion naturalness. Regardless of which of the two types of explanation is correct, robots with human-like appearance need to move their body in a human-like manner to avoid falling into the uncanny valley.

Today, certain realistic humanoid robots, referred to as *androids*, look similar to humans on the surface, and their appearance does not cause an uncanny feeling; some people do not even recognize them as robots at the first glance^8^. A representative example of an android with nearly human-like appearance is Geminoid F (developed by Osaka University and Advanced Telecommunications Research Institute International). The appearance of Geminoid F is indistinguishable from that of its human model, and it is capable of fine facial movement to implement realistic human-like movement^8,9^.

However, although the android is almost comparable to a human in appearance and movement, its movement is still slightly jerky and unnatural, due to the limitations of its joint system and actuators. Patients with motor symptoms (e.g., cogwheel rigidity) often exhibit slightly jerky movements that are caused by an impairment of the extrapyramidal system. The extrapyramidal system is known to automatically make our voluntary movement smooth and natural^10^. To achieve smooth movement, the extrapyramidal system must be responsive to mismatches between an ideally natural movement and the present state of body posture, where information on the latter is obtained via sensory inputs including visual observation. Since state-of-the-art androids and patients with motor symptoms caused by an impairment of the extrapyramidal system show similar, slightly unnatural movements, we hypothesized that activation in the extrapyramidal system increases when an observer sees slightly unnatural movements of an android. This hypothesis pertains to the neural mechanism of uncanny feelings about human-like robots.

**Figure 1:**
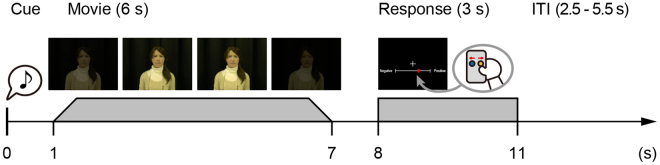
Trial sequence. Each trial started with a white fixation cross for 1 s accompanied by a short beep, and then a movie was presented in the centre of the screen for 6 s (Supplementary Video 1). Following a 1 s blank screen after the movie, a computer-based visual analogue scale (VAS) was presented on a 400-pixel horizontal line. In the response period, participants indicated their perceived emotional valence (negative-positive) of the movie within 3 s using a red cursor controlled by two buttons with their right thumb. The inter-trial interval was jittered from 2.5 to 5.5 s (mean interval of 4 s). Each participant performed 72 trials in a pseudo-randomized order.

To verify the hypothesis, we proceeded as follows: first, we recorded several movies of each of Geminoid F and its actual human model. Their differences in appearance were already minimal, and their differences in movement were minimized by asking the human model to act like the android. The human model tried to mimic the motions of the android as much as possible. She did well at controlling her facial expressions and the timing of motion, but her movements were slightly more natural than the android. This allowed us to investigate what differences a slight degree of motion unnaturalness would make to feelings about the android. Second, we used an fMRI to investigate what brain region activates when participants observed the android’s and the human’s movements in the movies where they were only slightly different in naturalness. During a scan, the participants were presented with the movies of the android (Supplementary Video 1) and the human model, and they were asked to focus on and rate the perceived emotional valence of both agents’ facial expressions (Fig. 1). The emotional valence-rating task was introduced for the purpose of preventing the participants from attending directly to differences in motion naturalness and related features between the android and the human model. The task consisted in judging the emotional valence of both agents in accordance with three pre-defined categories of facial expressions (positive, neutral, and negative). After the scan, the participants were presented with the same movies and asked to answer a questionnaire regarding the motion naturalness of the android and the human model.

## Results

**Figure 2:**
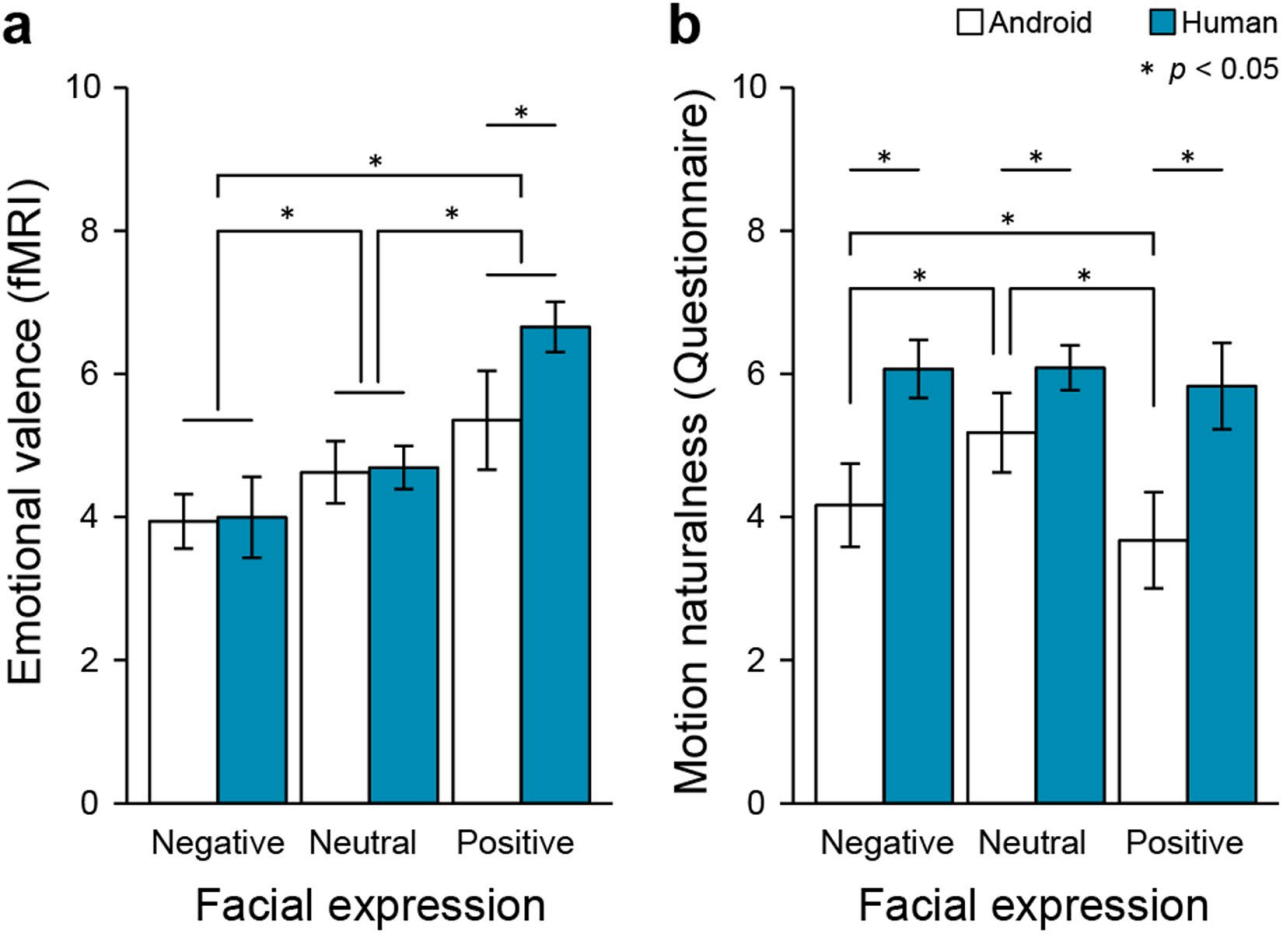
Results of the emotional valence task performed during the scan and the motion naturalness questionnaire performed after the scan. Each error bar represents the standard error of the mean.

Figure 2 shows the mean motion naturalness according to the post-scan questionnaire and the mean emotional valence during the MRI scans. We conducted a two-way repeated measures ANOVA on motion naturalness, and the results showed that the main effects of agent (Android, Human) [*F*_(1,13)_ = 89.24, *p* < 0.001], and facial expressions (Negative, Natural, Positive) [*F*_(2,26)_ = 8.53, *p* < 0.001], and their interaction [*F*_(2,26)_ = 9.89, *p* < 0.001], were significant. The results of multiple comparisons showed that there were significant differences between the Human and Android at all levels of facial expression, with the Android showing reduced motion naturalness than the Human. We then applied the same analysis to the results for emotional valence. The main effects of facial expressions [*F*_(2,26)_ = 46.29, *p* < 0.001] and their interactions [*F*_(2,26)_ = 19.04, *p* < 0.001] were significant; however, the main effect of agent was not significant [*F*_(1,13)_ = 3.13, *p* = 0.10]. These results indicated that the participants perceived the positive facial expressions of the human to be more pleasant than they did those of the android [*p* < 0.001].

**Figure 3:**
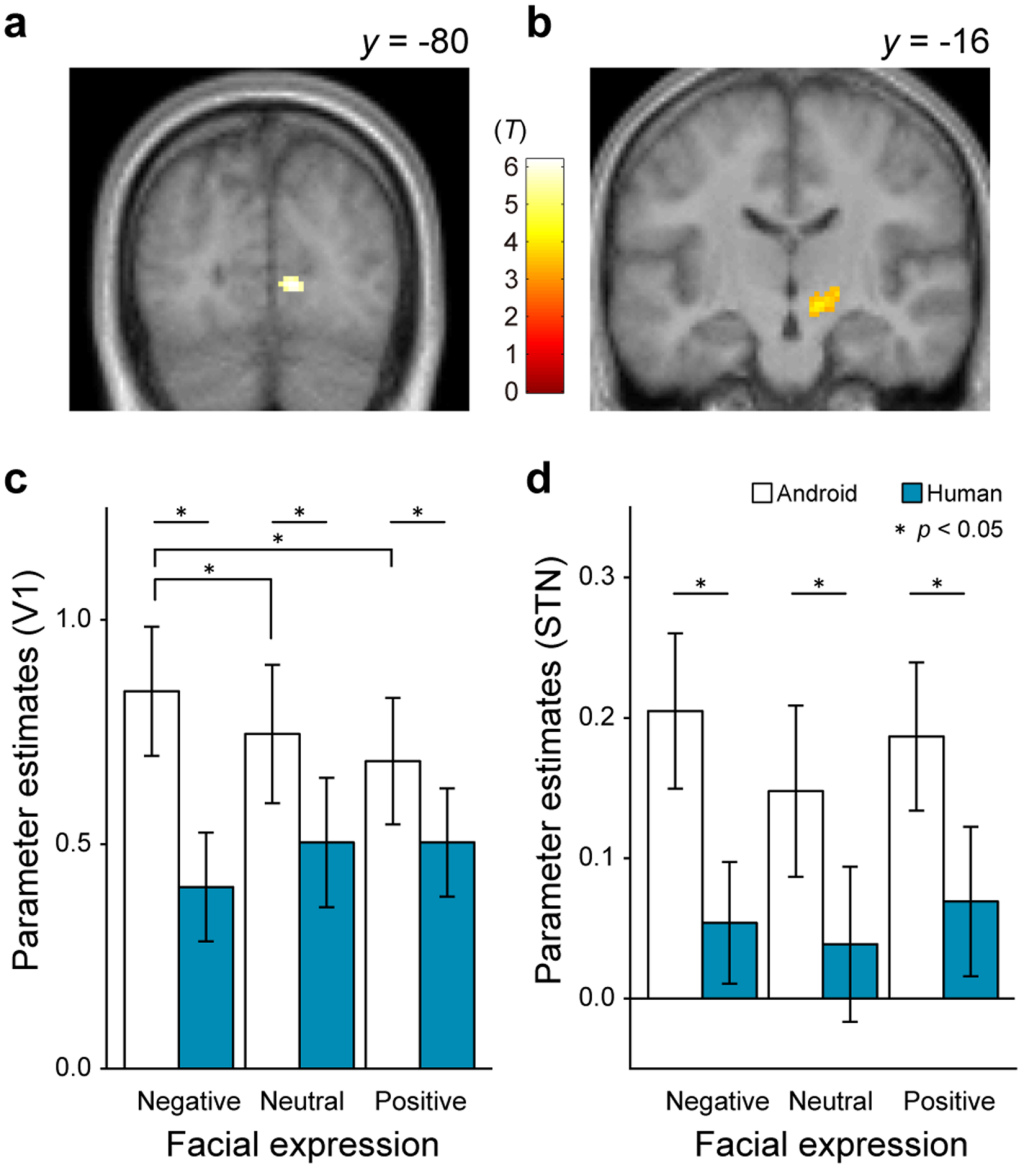
Activated areas (Android >Human, *p* < 0.05, FWE corrected): primary visual cortex (V1, **a**); and subthalamic nucleus (STN: *p* < 0.05, small-volume corrected (**b**). Parameter estimates at the peak voxel in V1 (**c**) and STN (**d**) are also shown. Each error bar represents the standard error of the mean.

Table 1 and Fig. 3 show the regions of the brain that were activated while the movies were played. The bilateral primary visual cortex (V1) was significantly activated under the Android condition. Furthermore, activation in the right STN was observed after small volume corrections under the Android condition. Nevertheless, activation of a brain cluster was not observed under the Human vs. Android comparison. The parameter estimates were extracted from the peak voxel in V1 (Fig. 3c) and STN (Fig. 3d). The result of a two-way repeated measures ANOVA on the parameter estimates in V1 was that the main effects of agent [*F*_(1,13)_ = 37.25, *p* < 0.001] and interaction [*F*_(2,26)_ = 4.31, *p* = 0.02] were significant. The result of a simple main effect analysis (Human vs. Android in each facial expression) indicated that the android’s movements enhanced the activation in V1 for each facial expression [*p*s < 0.05]. Within the factor of facial expression, the result of multiple comparisons using Shaffer’s modified Bonferroni procedure indicated that the negative facial expression of the android activated V1 more strongly than did its neutral [*p* = 0.03] or positive expression [*p* = 0.02]. The main effect of facial expressions was not significant [*F*_(2,26)_ = 0.20, *p* = 0.82]. The results in STN were that the main effect of agent was significant [*F*_(1,13)_ = 37.25, *p* < 0.001] and that the main effects of facial expressions [*F*_(2,26)_ = 0.60, *p* = 0.55] and their interaction [*F*_(2,26)_ = 0.15, *p* = 0.86] were not significant. The results implied that activation in STN was stronger under the Android condition than the Human condition, and it remains invariant with regard to facial expressions.

**Table 1:** Activated brain regions for each comparison.

Activation	L/R	BA	*k*E	Voxel level			MNI coordinates		
				*p* (FWE)	*T*	*Z*	*x*	*y*	*z*
**Android >Human (whole brain analysis)**									
Calcarine sulcus	R	17	34	0.001	6.17	5.55	8	−80	0
**Android >Human (with SVC)**									
Subthalamic nucleus	R	—	25	0.041	4.08	3.87	10	−16	−8
**Human >Android**									
No suprathreshold voxels									

The statistical threshold was set at *p* < 0.05, which was FWE corrected at the voxel level. Based on prior knowledge, we applied a small volume correction within an anatomical mask comprising the basal ganglia.

## Discussion

We found that the motion naturalness of the android was consistently lower than that of the human agent for each facial expression (Fig. 2b). In addition, we confirmed that the participants correctly discriminate the facial expressions of three categories for both agents and that the human agent had an enhancing effect on positive facial expressions, compared with the android (Fig. 2a). It follows from these behavioural results that a decrease in motion naturalness does not always induce unpleasant feelings. The results are consistent with the past finding that when an android moves its eyes unnaturally and slightly jerkily, its smile still appears to be a false smile, and the appearance of a false smile is induced by imperfect movement of the orbicularis oculi^11^.

Based on the results of fMRI data, we found significant activation in the right STN under the Android condition. The STN is part of the extrapyramidal system and plays an important role in motor control^12,13^. The extrapyramidal system, including the hyperdirect pathway that bypasses the striatum^14,15^, reduces cortical overactivity and increases voluntary movement smoothness. The STN is involved in the process of impairing dopaminergic neurons in the substantia nigra in Parkinson’s disease patients, and deep brain stimulation to the STN dramatically improves akinesia or rigidity in these patients. This effect is likely caused by a restoration of inhibitory output to the striato-thalamo-cortical loop under stimulation^16,17^. Our study attests to commonalities between the movements of the android and a Parkinson’s disease patient. First, nine participants reported that the android moved her head rigidly (Supplementary movie 1). Second, the valence of positive facial expressions of the android was lower that of the human agent (Fig. 2a). These results indicate that the android’s movements were rigid and akinesic in a comparable way to the movements of a patient with mild Parkinson’s disease. We did not find any significant voxel difference between positive facial expressions of the two agents, and therefore the neural basis of the positive effect of human movements is left for further research.

Our results, then, lead to an important question: why was the STN activated when the participants observed unnatural movement? The answer is related to the important role played by the STN in one’s own voluntary movement^18,19^. According to recent studies, observations of grasping^20^ and wrist extension^21^ movements in others activate the STN, and STN activation is dynamically synchronize with sensorimotor cortex and basal ganglia activation after movement^22^. These studies suggest that STN activation could encode motor information, including motor imagery^23^, as well as visual information related to action observations. In the present study, we found that STN activation increased when unnatural movement was observed in the android. The STN also plays a role in monitoring and evaluating movement errors^22^ as well as in voluntary movement. Our results suggest that the STN implicitly monitors motion naturalness through visual feedback. Thus, when an android moves in an unnatural manner, the STN may detect this unnaturalness because an error signal results from a mismatch between a visual input and an internal model for smooth movement.

In addition to the STN, the primary visual cortex was activated when the participants observed unnatural movements. A possible cause of increased activation in the primary visual cortex is an attentional effect. Many cortical and subcortical regions give rise to projections to V1; in particular, modulatory feedback from the higher visual areas can regulate activation in V1^24^. Moreover, activation in V1 increases when people observe a low-intentionality-involving animation of a moving object^25^. Thus, if the android’s unnatural movement alleviates perception of intentionality or animacy, it may activate V1. In contrast to the STN (Fig. 3d), activation in V1 was increased by the android’s negative facial expressions (Fig. 3c). Although V1 tends to respond to an unpleasant stimulus but not to a positive stimulus^26^, the human’s negative facial expressions did not affect the increase in activation of V1. This is probably because emotional salience had an effect on attention^27^. If the android’s movement involves high attentional demands, the android’s negative facial expressions may increase activation in V1 to a further extent. A possibility remains that activation in V1 itself may be a source of uncanny feelings. According to one ERP study on the uncanny valley^28^, animated face stimuli elicited the N170 component, whereas realistic face stimuli activated a more interior part of the occipital lobe. The N170 is associated with face perception, and it is estimated in the primary visual cortex including V1. Moreover, the N170 is enhanced by a negative facial expression to a greater degree than it is by a neutral or positive expression. Most V1 neurons respond to primitive visual features of stimuli, such as orientation or luminance. Therefore, our results are consistent with the possibility that, in addition to the detection of motion naturalness in the STN, another source of an agent’s human-likeness is the detection of the agent’s primitive features in V1, e.g., joint angles, moving edges, and their accelerations.

Today, artificial social agents have already been used in the medical and health-care fields (e.g., a realistic android is now employed as a trainer for improving social skills for adults with autism spectrum disorder^29^). It is important for such social agents in use to minimize uncanny feelings with the installation of legitimate kinetic and visual features. However, a critical issue is to identify the kinetic and visual features that are responsible for uncanny feelings. It is markedly difficult to improve an android with highly complex structure through trial and error. The findings of the neural mechanisms of uncanny feelings should guide the development of human-friendly artificial social agents in efficient and practical ways. The results of the current study show that observation of an android’s unnatural movements result in activation in the STN and V1. The making of a more human-friendly android, then, requires that it be designed to have the kinetic features that suppress activation in the STN and the primitive visual features that induce a moderate activation in V1, such as proper joint angles and angular velocities.

## Methods

### Participants

Fourteen healthy right-handed volunteers (3 females and 11 males, ages 21–26) participated in this study. All of the participants had normal or corrected-to-normal vision. None of the participants had a history of neurological or psychiatric disorders or specific experience with a humanoid-robot. Written informed consent was obtained from all participants. The experiment was conducted in accordance with the ethical guidelines of the Declaration of Helsinki, and all methodology was approved by the Ethics Committee of Advanced Telecommunications Research Institute International (ATR).

### Stimuli

The stimuli were movies of emotional facial expressions performed by two agents: the android (Geminoid F, Supplementary Video 1) and the human model of the android. Thirty-six facial expressions in 3 categories were classified as negative (12 patterns), neutral (10 patterns) and positive (14 patterns) with different valences. All of the facial expression patterns were filmed by the experimenters (72 movies in total). Each movie started in a neutral state and dynamically changed to show a certain valence of emotion within 6 s. We applied a 0.5 s fade-in period and a 0.5 s fade-out period at the beginning and end of each movie. The outfit and background were the same for all movies, which were recorded in 30 fps, encoded with the DivX codec, and resized to 640 × 480 pixels. The movies were produced using the following procedure: 36 facial expressions performed by the human agent were recorded; the android’s motion mimicking each of the human’s expressions were programmed, and 36 videos of the android’s facial expressions were recorded; and then the human agent was asked to mimic the android’s motions, and 36 corresponding videos of the human’s facial expressions were recorded. This last step was important because the android’s range of motion was narrower than that of the human’s.

### fMRI experiment

Figure 1 shows the experimental procedure. Each participant watched all of the 72 movies and indicated his/her perceived emotional valence for each movie while in a MRI scanner. The emotional valence task was only indirectly related to motion naturalness; therefore, the participants were asked not to pay particular attention to the agents’ motions. Each trial started with a white fixation cross, which was presented for 1 s and accompanied by a short beep, and then a movie was displayed in the centre of the screen for 6 s. Following a 1 s blank screen after the movie, a computer-based visual analogue scale (VAS) was presented on a 400 pixel horizontal line. In the response period, the participant indicated his or her perceived emotional valence (negative–positive) of the movie within 3 s by controlling a red cursor, which was moved by pushing two buttons with his or her right thumb. The inter-trial interval was jittered from 2.5 to 5.5 s (mean interval of 4 s). Each participant performed 72 trials in a pseudo-randomized order. After the scan, the participants answered a questionnaire that included questions such as “Did you know Geminoid F?” and “Could you discriminate the android (Geminoid F) from a human?” The questionnaire also included questions related to how naturally both agents moved in each movie, and the perceived valence was measured by printed VASs of 100 mm.

The fMRI experiment was conducted in a 3-T MRI scanner (Verio, Siemens, Erlangen, Germany). A forehead strap was used to reduce the participant’s head motion, and functional images were taken with a gradient echo-planar pulse sequence in an interleaved order (TR = 3000 ms, TE = 27 ms; flip angle = 80°, voxel size = 3 mm × 3 mm × 3 mm, and 49 axial slices). Subsequently, anatomical T1-weighted images (MPRAGE sequence, TR = 2250 ms, TE = 3.06 ms, flip angle = 9°, field of view = 256 × 256 mm, voxel size = 1 × 1 × 1 mm, and 208 sagittal slices) were collected.

### Image processing and analysis

Image processing and analysis were performed using SPM8 (Welcome Department of Cognitive Neurology, London, UK) running on MATLAB (MathWorks Inc., Shervorn, MA, USA). First, we conducted slice acquisition timing correction on the functional images, and the images were then realigned to the mean image to correct for head movement. Then, the T1-weighted anatomical image and the realigned functional images were normalized to the Montreal Neurological Institute MNI (MNI) space using the T1 and EPI template images included in SPM8, respectively. The normalized functional images were smoothed with an isotropic Gaussian kernel with an 8 mm full width-half maximum. Low-frequency noise was removed using a high-pass filter (time constant of 128 s).

Individual analyses were performed with a fixed effect model. Statistical parametric maps were calculated to identify voxels with event-related BOLD signal changes using a general linear model (GLM). Six regressors represented the functions of the observation period (Geminoid, Human × Negative, Neutral, Positive), and a regressor of the response to the VAS period was entered. Each event was convolved with a canonical hemodynamic response function (HRF) to provide regressors for the GLM. Head movement parameters calculated in the realignment step were included in this model as regressors of no interest. Contrast images were taken to the second-level *t*-test to produce statistical maps at the group level using the random effect model. The statistical threshold was set to *p* < 0.05 (FWE corrected). Furthermore, we conducted an analysis using small volume corrections (*p* < 0.05, FWE corrected) within an anatomical mask of the basal ganglia, including the subthalamic nucleus, red nucleus, globus pallidus, putamen, and caudate. This mask was produced using WFU_PickAtlas^30,31^. Parameter estimates of signal intensity were extracted from the peak voxels in V1 and the STN using the MarsBaR Toolbox^32^.

## Electronic supplementary material

Supplementary video
